# Supplementary material for: Generative approaches to kinetic parameter inference in metabolic networks via latent space exploration
Source: Nat Commun. 2026 Apr 20;17:5430. doi: 10.1038/s41467-026-72184-3 (PMC13280403; doi:10.1038/s41467-026-72184-3)
Supplement: Supplementary file 3 — Transparent Peer Review file [file 41467_2026_72184_MOESM3_ESM.pdf]

nature portfolio

Peer Review File

Generative Approaches to Kinetic Parameter Inference in Metabolic Networks via Latent Space Exploration

Corresponding Author: Dr Ljubisa Miskovic

This file contains all reviewer reports in order by version, followed by all author rebuttals in order by version.

Version 0:

Reviewer comments:

Reviewer #1

(Remarks to the Author)
The authors present a framework that enables repurposing generative neural networks (GNNs) trained to produce kinetic models of metabolic networks for different physiological contexts. The work builds upon their previous tools called REKINDLE and RENAISSANCE, which utilize GNNs for efficient parameter generation. The manuscript demonstrates the approach through three case studies in E. coli, specifically for (1) fine-tuning dynamic response times in aerobic metabolism, (2) identifying enzymatic bottlenecks that control response speed, and (3) adapting neural networks trained for one context to describe anaerobic metabolism dynamics. The authors show that by working with the latent space of trained generators, they can systematically adjust model properties and identify key parameters governing system dynamics without retraining. The approach significantly improves upon traditional sampling-based methods, potentially enabling high-throughput dynamic studies across multiple physiologies. Overall, the paper presents a novel approach to repurpose trained generative neural networks by manipulating their latent space, offering an efficient alternative to retraining models for different physiological contexts.

Major comments:
1. The authors selected the generator(s) with the highest incidence of valid models for their analyses. This approach may lead to bias and/or limit the generalizability of the findings, especially when a low number of statistical repeats were performed. The manuscript would benefit from a discussion, or even better a demonstration, of how results might vary when using different generators or an ensemble approach. Are there any guidelines on how many statistical repeats would be sufficient?

2. Perturbing individual latent axes to find significant features and then assessing how τ_max depends on top latent variables is in essence equivalent to performing sensitivity analysis in latent space rather than parameter space. Since the biochemical interpretation ultimately requires mapping back to the actual kinetic parameters, how does this approach compare to traditional GSA methods like Sobol indices in terms of computational efficiency, parameter identification accuracy, and handling of parameter interactions? For example, does latent space exploration require fewer model evaluations? Given the same computational budget, how well do both approaches identify key parameters? Besides, how does the latent space approach capture parameter interactions compared to variance-based GSA methods?

3. The authors demonstrate their framework using E. coli, which has a well-characterized metabolism. How does the performance depend on the knowledge of an organism's metabolism? Have the authors attempted their approach on less characterized metabolic networks, with more limited kinetic measurements or more poorly defined network topologies? To what extent does the quality of the latent space representation depend on the comprehensiveness of available metabolic data/knowledge? Finally, what (minimum) level of metabolic characterization would be required for their generative approach to produce biologically meaningful results? A discussion on this topic would improve the manuscript, specifically addressing the broader applicability of the proposed approach beyond model organisms.

(Remarks on code availability)
It would be useful to provide the generator(s) that the authors trained and used in the study.

Reviewer #2

(Remarks to the Author)
The paper by Choudhury et al. presents a machine learning framework for studying kinetic models of biological processes and adapting results obtained for one system to other physiological conditions. They authors utilize the latent space of generative neural networks to investigate important kinetic parameters and dynamic bottlenecks in metabolic networks, and they demonstrate that a generative neural network trained in one context can be repurposed to build kinetic models in another, without retraining from scratch.

The paper is clearly written, consists of an interesting central idea, and highlights useful aspects of the method. However, I view it as a modest extension of the authors' recent work on generative ML-based parameterization techniques (REKINDLE and RENAISSANCE). As detailed below, my main concern is that the paper does not explore the potential of repurposing generative neural networks to new contexts in sufficient depth to justify publication in Nature Communications.

Major concerns

1. I agree with the authors that the "field would greatly benefit from the ability to repurpose generative neural networks designed for a specific physiology and/or organism to other studies." However, my main concern is that the paper does not deeply explore this potential. The authors present only a single test case involving E. coli. To merit publication in a high-impact journal, the contribution should extend beyond a single proof of concept and more directly address the broader applicability of the approach. For example, for a generator trained for an organism under particular conditions, how different can the new conditions be? Can it work for different organisms? What determines the limits of applicability?

2. How do the authors choose the dimensionality of the latent space? How does this choice impact the conclusions of this paper?

3. In the discussion of Figure 3, the authors state that the results demonstrate "that latent space manipulation enables direct identification of the key parameters governing a model's dynamic properties." However, it is not clear that the sampling is systematic. They later state that "the transformation generates structured datasets that are well-suited for statistical analysis." The meaning of this statement is not clear to me.

In general, could the latent space sampling miss kinetic regimes that might reveal other important parameters? Could correlations between kinetic parameters built into the sampling pose issues with respect to statistical analysis?

4. I am not fully convinced by the arguments the authors make surrounding the results in Figure 4. The authors identify kinetic parameters associated with PDH, GAPDH, and CS as the primary determinants of τ_{max} across the network. However, they are not the highest "hits" in steady state 1, and other parameters have high occurrence in individual steady states.

Minor concerns

- The methods related to sampling "four different thermodynamically feasible reference profiles of fluxes and metabolite concentrations using Thermodynamic Flux Balance Analysis" were not clear. The text in the Methods section seemed incomplete and some of it read as if generated by an LLM (referring to a "study titled" did not make sense).

- Figure 5c: "Left" and "right" are switched. There is a reference to text in Figure 5d that is not present.

(Remarks on code availability)

Version 1:

Reviewer comments:

Reviewer #1

(Remarks to the Author)
Thank you for addressing my comments.

Additional minor comment: Scripts for Figure 6 on the GitHub page was still labelled Figure 5.

(Remarks on code availability)
Codes have README file.
I did not try to run the code as I do not have GPU capability on my machine.

Reviewer #2

(Remarks to the Author)
The authors have improved the manuscript in revision and have adequately addressed my concerns.

(Remarks on code availability)

Open Access This Peer Review File is licensed under a Creative Commons Attribution 4.0 International License, which permits use, sharing, adaptation, distribution and reproduction in any medium or format, as long as you give appropriate credit to the original author(s) and the source, provide a link to the Creative Commons license, and indicate if changes were made.
In cases where reviewers are anonymous, credit should be given to 'Anonymous Referee' and the source.
The images or other third party material in this Peer Review File are included in the article's Creative Commons license, unless indicated otherwise in a credit line to the material. If material is not included in the article's Creative Commons license and your intended use is not permitted by statutory regulation or exceeds the permitted use, you will need to obtain permission directly from the copyright holder.
To view a copy of this license, visit https://creativecommons.org/licenses/by/4.0/

Authors response to the reviewers' comments

Title: Generative Approaches to Kinetic Parameter Inference in Metabolic Networks via Latent Space Exploration
Tracking #: NCOMMS-25-56564
Authors: Choudhury et al.

We thank reviewers for their insightful and constructive comments, which have substantially improved the quality, clarity, and scope of our manuscript. In response, we performed several new computational studies and expanded key methodological explanations. Briefly, the revised manuscript now includes:

(i) A new study applying our framework to *S. cerevisiae*. This addresses the reviewers' concerns about generalizability to other organisms and the robustness of our findings across generators with different incidences of desired properties. These new results demonstrate that our procedure remains effective across a broad range of generators and in a eukaryotic metabolic network.
(ii) A global sensitivity analysis (GSA) in which we computed first-order Sobol indices and compared them directly to the sensitivities identified through latent-space exploration. This analysis clarifies the relationship between latent-space perturbations and established GSA methodologies.

We have revised the main manuscript to incorporate the insights from these new studies. In particular, we added a dedicated Results subsection describing the *S. cerevisiae* application together with a new figure illustrating the corresponding latent space analysis, and a new Supplementary Note 3, which discusses and compares global sensitivity analysis with latent space exploration. We have also extended the Methods section with several subsections related to the S. cerevisiae application. Moreover, we have included additional details as requested by the reviewers to facilitate a comprehensive understanding of the presented material.

Our point-by-point responses to the reviewers' comments are provided below in blue.

Finally, the revised manuscript is available in two versions: a clean copy and a version with all changes highlighted in blue.

REVIEWER COMMENTS

Reviewer #1 (Remarks to the Author):

The authors present a framework that enables repurposing generative neural networks (GNNs) trained to produce kinetic models of metabolic networks for different physiological contexts. The work builds upon their previous tools called REKINDLE and RENAISSANCE, which utilize GNNs for efficient parameter generation. The manuscript demonstrates the approach through three case studies in E. coli, specifically for (1) fine-tuning dynamic response times in aerobic metabolism, (2) identifying enzymatic bottlenecks that control response speed, and (3) adapting neural networks trained for one context to describe anaerobic metabolism dynamics. The authors show that by working with the latent space of trained generators, they can systematically adjust model properties and identify key parameters governing system dynamics without retraining. The approach significantly improves upon traditional sampling-based methods, potentially enabling high-throughput dynamic studies across multiple physiologies. Overall, the paper presents a novel approach to repurpose trained generative neural networks by manipulating their latent space, offering an efficient alternative to retraining models for different physiological contexts.

Major comments:
1. The authors selected the generator(s) with the highest incidence of valid models for their analyses. This approach may lead to bias and/or limit the generalizability of the findings, especially when a low number of statistical repeats were performed. The manuscript would benefit from a discussion, or even better a demonstration, of how results might vary when using different generators or an ensemble approach. Are there any guidelines on how many statistical repeats would be sufficient?
As noted in the preamble to our detailed responses, to address this concern, we designed an additional study in which the proposed framework is applied to *S. cerevisiae* using three generators sampled at different stages of training and across distinct regions of the latent space. This analysis

explicitly examines the robustness and generalizability of our findings and demonstrates that the qualitative conclusions are preserved across generators with varying incidences of valid models, thereby illustrating the approach's versatility.

For clarity, in our original studies, we selected generators with high (though not necessarily the highest) incidences of valid models primarily for demonstration, convenience, and interpretability. Importantly, the underlying methodology of latent space manipulation is independent of the absolute incidence of valid models produced by a given generator. We note, for example, that in Figure 4 we already employed multiple generators derived from different steady states to support our conclusions.

We do not expect identical quantitative outcomes when using generators with substantially lower incidences, owing to the highly nonlinear mapping learned by neural networks and the intrinsically rugged fitness landscape of the parametric space. In this context, generators with higher incidences provide a more reliable basis for analysis, as they reflect a more stable training regime and a more robust correspondence between latent and parametric spaces. This observation is consistent with the results of the newly added *S. cerevisiae* study.

Finally, to facilitate transparency and reproducibility, all generators produced during training are made publicly available in the supplementary dataset and can be readily incorporated into the analysis scripts provided in the public code repository, enabling direct comparison across generators and statistical repeats.

2. Perturbing individual latent axes to find significant features and then assessing how τ_max depends on top latent variables is in essence equivalent to performing sensitivity analysis in latent space rather than parameter space. Since the biochemical interpretation ultimately requires mapping back to the actual kinetic parameters, how does this approach compare to traditional GSA methods like Sobol indices in terms of computational efficiency, parameter identification accuracy, and handling of parameter interactions? For example, does latent space exploration require fewer model evaluations? Given the same computational budget, how well do both approaches identify key parameters? Besides, how does the latent space approach capture parameter interactions compared to variance-based GSA methods?

The reviewer is right in commenting that any latent space perturbation is eventually mapped back to the parametric space for integration into the model and subsequent calculations. However, a key distinction is that manipulation of individual latent dimensions simultaneously perturbs all kinetic parameters in a structured, coordinated manner, which is substantially more efficient than perturbing parameters individually, in pairs, or at higher orders to assess their effects on the dominant time constants (τ_{max}).

Since typical metabolic models comprise a large number of kinetic parameters, exhaustively perturbing individual parameters to evaluate their contributions to output variance (e.g., τ_{max}) quickly becomes impractical, particularly when higher-order interactions are considered. For example, the *E. coli* model used in the first three studies is one of the most established kinetic models in the community and contains D = 258 kinetic parameters. Considering a base sample size of N = 256 (relatively coarse-grained) or N = 1024 (high precision), computing first-order Sobol indices using Saltelli' sampling scheme[1,2] would require approximately 66.3k and 265.2k model evaluations, respectively (total runs = N × (D + 1)). Including second-order and total-order indices increases the required evaluations to approximately 66.6k and 266.2k (total runs = N × (D + 2)). More generally, including Mth-order interaction effects increases the computational cost linearly with M, so that the total number of runs equals N × (D + M), making exhaustive variance-based sensitivity analysis increasingly impractical for large-scale kinetic models.

By contrast, the results presented in Figure 2 required only 1,681 model evaluations using latent space–based sensitivity analysis. Thus, latent space exploration provides a significantly more efficient and scalable alternative to comprehensive exploration of the parametric space, requiring orders of magnitude fewer model evaluations to capture comparable variance in system-level outputs. This efficiency advantage is particularly relevant for large-scale kinetic models, where traditional GSA rapidly becomes computationally prohibitive.

Additionally, kinetic parameters in biochemical networks have been shown to be sloppy with respect to system dynamics, meaning that many parameters can vary substantially without strongly affecting macroscopic behavior. As a consequence, individual parameter perturbations are difficult to interpret

and require careful, parameter-specific sensitivity bounds, which must be specified *a priori* for variance-based GSA methods.

Nevertheless, in direct response to the reviewer's question, we computed the first-order Sobol indices for all 258 kinetic parameters of the *E. coli* model used in the first three studies, using N = 1024 to obtain a high-precision sensitivity estimate. The distribution of the resulting Sobol indices is shown below.

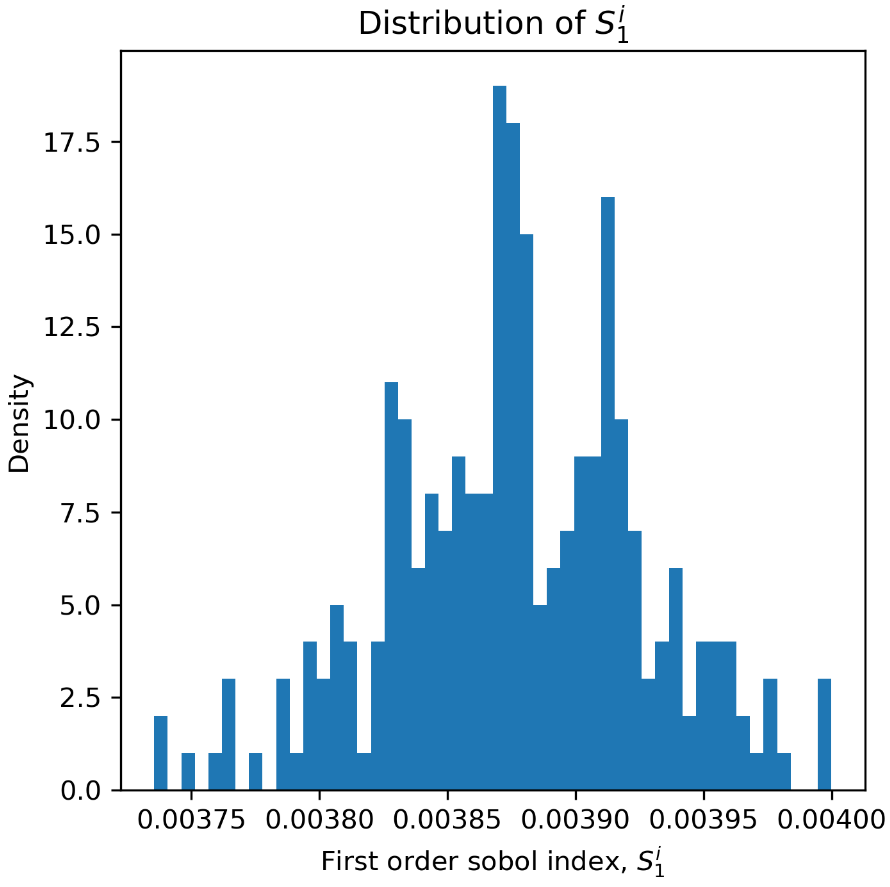

Figure: Distribution of first-order Sobol indices obtained.

We observe that all Sobol indices fall within a narrow range between 0.0037 and 0.004, corresponding to sensitivities of approximately 3.5–4%, with no discernible outliers dominating the system's dynamics. In contrast, latent space–based analysis enables the identification of specific kinetic parameters whose coordinated variation strongly affects dynamic behavior, despite their weak individual Sobol sensitivities.

Finally, because latent space exploration perturbs all parameters simultaneously, it naturally captures higher-order parameter interactions without requiring explicit enumeration, unlike variance-based GSA. Using the same statistical framework described in the manuscript, we analyzed parameter and enzyme co-occurrence statistics (see Supplementary Note 1). While no statistically significant interaction clusters were detected in this case, the framework itself remains applicable to interaction analysis without additional computational burden.

We have incorporated this study into the revised Supporting Information as Supplementary Note 3.

3. The authors demonstrate their framework using *E. coli*, which has a well-characterized metabolism. How does the performance depend on the knowledge of an organism's metabolism? Have the authors attempted their approach on less characterized metabolic networks, with more limited kinetic

measurements or more poorly defined network topologies? To what extent does the quality of the latent space representation depend on the comprehensiveness of available metabolic data/knowledge? Finally, what (minimum) level of metabolic characterization would be required for their generative approach to produce biologically meaningful results? A discussion on this topic would improve the manuscript, specifically addressing the broader applicability of the proposed approach beyond model organisms.

Both REKINDLE and RENAISSANCE require predefined network stoichiometry, kinetic rate laws, and reference steady-state fluxes and metabolite concentration profiles in order to generate biologically consistent kinetic parameter sets. Because the framework presented in this manuscript operates on top of these trained generative models, it necessarily inherits the same prerequisites and limitations.

Importantly, however, the newly added *S. cerevisiae* study directly addresses the reviewer's question regarding dependence on organism-specific metabolic knowledge. Compared to *E. coli*, *S. cerevisiae* metabolism is less comprehensively characterized at the kinetic level, with sparser experimental measurements and greater uncertainty in parameterization. Despite this, our results demonstrate that latent space exploration remains effective in modulating system dynamics across multiple generators, training stages, and regions of latent space, indicating that the approach's performance does not critically rely on exhaustive kinetic knowledge at the level of individual parameters.

This observation highlights a key advantage of our framework: while detailed kinetic measurements improve realism, the latent space representation itself captures system-level dynamical structure that emerges from network topology, stoichiometric constraints, and steady-state feasibility, rather than from precise identification of individual kinetic constants. In the *S. cerevisiae* case, even with increased uncertainty in kinetic parameters, the latent space remained sufficiently structured to enable reproducible modulation of dominant time scales and stability properties.

With respect to broader applicability, the quality of the latent space representation depends primarily on the consistency and completeness of the metabolic network definition and the availability of at least one feasible steady state, rather than on comprehensive kinetic parameter datasets. As long as the stoichiometry is well defined, plausible kinetic rate laws are assigned, and steady-state fluxes and metabolite concentrations can be estimated (e.g., from constraint-based models or omics-informed reconstructions), the generative approach can be trained and meaningfully explored.

Consequently, the minimum level of metabolic characterization required for the proposed framework to yield biologically meaningful insights includes:
(i) a curated stoichiometric network,
(ii) assignment of generic but biochemically plausible kinetic mechanisms, and
(iii) one or more feasible steady-state operating points.
High-resolution kinetic measurements, while beneficial, are not strictly necessary.

Taken together, the *S. cerevisiae* study demonstrates that latent space exploration is not restricted to highly curated model organisms, but can be applied to organisms with moderate or incomplete kinetic knowledge, provided that the underlying metabolic network structure is available. This substantially broadens the applicability of the proposed framework to non-model organisms and emerging genome-scale reconstructions, addressing the reviewer's concern regarding generalizability.

We have incorporated this discussion into the revised Discussion section, where we explicitly address the dependence of the proposed framework on metabolic knowledge, its applicability to less characterized organisms, and the minimum data requirements for meaningful use.

Remarks on code availability:
It would be useful to provide the generator(s) that the authors trained and used in the study.
All generators used in the manuscript, as well as unused generators from the training process, are available in the publicly available code repository (please refer to Data and Code Availability sections).

Reviewer #2 (Remarks to the Author):

The paper by Choudhury et al. presents a machine learning framework for studying kinetic models of biological processes and adapting results obtained for one system to other physiological conditions. They authors utilize the latent space of generative neural networks to investigate important kinetic parameters and dynamic bottlenecks in metabolic networks, and they demonstrate that a generative

neural network trained in one context can be repurposed to build kinetic models in another, without retraining from scratch.

The paper is clearly written, consists of an interesting central idea, and highlights useful aspects of the method. However, I view it as a modest extension of the authors' recent work on generative ML-based parameterization techniques (REKINDLE and RENAISSANCE). As detailed below, my main concern is that the paper does not explore the potential of repurposing generative neural networks to new contexts in sufficient depth to justify publication in Nature Communications.

Major concerns

1. I agree with the authors that the "field would greatly benefit from the ability to repurpose generative neural networks designed for a specific physiology and/or organism to other studies." However, my main concern is that the paper does not deeply explore this potential. The authors present only a single test case involving E. coli. To merit publication in a high-impact journal, the contribution should extend beyond a single proof of concept and more directly address the broader applicability of the approach. For example, for a generator trained for an organism under particular conditions, how different can the new conditions be? Can it work for different organisms? What determines the limits of applicability?

We agree with the reviewer that demonstrating broader applicability beyond a single proof of concept is essential. In response to this concern, we have substantially extended the manuscript by adding a new study on *Saccharomyces cerevisiae*, thereby moving beyond a single *E. coli* test case and explicitly addressing generalizability of the approach to other organisms.

Generative methods such as REKINDLE and RENAISSANCE are model-agnostic in the sense that they can be trained to parameterize any metabolic network for which stoichiometry, kinetic rate laws, and at least one feasible steady-state operating point are available. The methodology introduced in this work operates on top of such trained generators and therefore inherits their prerequisites. Importantly, however, the added *S. cerevisiae* analysis demonstrates that latent space exploration remains effective even for an organism whose metabolism is less comprehensively characterized at the kinetic level than *E. coli*. Across multiple generators, training stages, and regions of latent space, we observe reproducible control over dynamic properties, indicating that the approach does not rely on organism-specific fine-tuning or exhaustive kinetic knowledge.

Regarding the limits of applicability, our results suggest that these are not determined by species identity per se, but rather by the consistency of the underlying metabolic model and the availability of feasible steady states used during generator training. Differences in physiological conditions or organisms are reflected as different regions of latent space, which can be systematically explored and repurposed without retraining the generator. However, we do not expect arbitrarily large extrapolations, such as radically altered network topology or incompatible steady states, to be captured reliably by a generator trained on a fundamentally different system. In this sense, the limits of applicability are set by the coverage of the training distribution and the structural assumptions encoded in the model, rather than by the latent space methodology itself.

These points are now addressed in the revised Discussion section, together with the newly added *S. cerevisiae* study, to clarify the scope and generality of the proposed approach.

2. How do the authors choose the dimensionality of the latent space? How does this choice impact the conclusions of this paper?

The dimensionality of the latent space for REKINDLE and RENAISSANCE was chosen according to recommended hyperparameter settings for GANs and Evolutionary Strategies, respectively, as established in the neural network and machine learning literature. We deliberately did not alter the latent dimensionality in order to remain consistent with these well-validated training configurations.

We agree with the reviewer that latent space dimensionality can, in principle, influence the behavior and expressiveness of generative models. In the context of this work, however, our primary conclusions, namely, the ability to systematically explore and repurpose generators via structured latent space manipulations, do not rely on a specific latent dimensionality choice. This is supported by the newly introduced *S. cerevisiae* study, which demonstrates that latent representations trained on different organisms retain robustness and interpretability despite differences in physiology and parameter uncertainty. Together, these results suggest that our conclusions are not critically sensitive

to the precise latent dimensionality, provided it lies within standard, well-performing ranges for these architectures.

3. In the discussion of Figure 3, the authors state that the results demonstrate “that latent space manipulation enables direct identification of the key parameters governing a model’s dynamic properties.” However, it is not clear that the sampling is systematic. They later state that “the transformation generates structured datasets that are well-suited for statistical analysis.” The meaning of this statement is not clear to me.
We assume that when the reviewer states ‘that it is not clear the sampling is systematic’, they are referring to the mapped sampling in the parametric space. The samples in the latent space are generated via simple linear transformations, as outlined in the manuscript, and lie on a regular grid in the multidimensional latent space. The reviewer is correct that this linearity is not preserved when mapped to the parametric space due to the highly nonlinear nature of the neural network.
Latent space transformations, even the simplest linear ones, change all kinetic parameters at once when mapped (Figure 2b). However, these changes are mostly smooth and monotonic (differentiable in the whole domain) when mapped to the parametric space, as seen clearly in Supplementary Figure 8. These smooth changes in the parametric regime are further evidenced in the high correlations/anti-correlations seen between any generated kinetic parameter and τ_{max} when latent axes are varied in small linear increments (Figure 3a, left), compared to when kinetic parameters are randomly sampled (Figure 3a, right). Thus, even though the mapping is nonlinear, linear transformations in the latent space generate smoothly varying kinetic parameters, which are more amenable to statistical analysis than randomly sampled kinetic parameters.

In general, could the latent space sampling miss kinetic regimes that might reveal other important parameters? Could correlations between kinetic parameters built into the sampling pose issues with respect to statistical analysis?
The sampling is dependent on the quality of mapping achieved between the latent space and parametric space during the training process. To ensure good training, proper practices and metric monitoring (convergence of discriminator accuracy to 50% for GANs, convergence of high incidences for evolutionary strategies) were implemented when training the neural networks (Supplementary Figure 3,4). Moreover, only generators with a relatively high incidence of relevant models were used for studies outlined in this manuscript, as they are a good indicator of the successful mapping. Additionally, our published studies show that REKINDLE and RENAISSANCE generate models with high output diversity and can reliably produce kinetic parameter sets with high variance. It is possible that a trained generator might have a mapping that excludes certain parametric regimes, but the checks outlined above ensure that the ‘potentially missed’ kinetic regimes are not statistically significant compared to what is captured in a mapping with a good training process.

Additionally, the reviewer is correct in commenting that kinetic parameters might be correlated with the system’s dynamics and amongst themselves spuriously due to the highly non-linear nature of the neural networks and the ODE system. We agree with the reviewer that spurious, or ‘artefact’ correlations affect statistical analyses. In all our analyses, highly correlated parameters were individually verified to determine whether they affected the system’s properties (dominant time constants) to the same degree. Those that did not affect the system properties were discarded from further analyses. Kinetic parameters with high correlation with the latent axes but low effect on the system’s dynamics (low causation) are sloppy. This has been thoroughly discussed in the results section pertaining to Figure 2. Kinetic parameters that exhibit spurious correlations with other kinetic parameters are not relevant, as high correlation and high causation with respect to the system dynamics are considered the necessary criteria for further statistical analysis.

4. I am not fully convinced by the arguments the authors make surrounding the results in Figure 4. The authors identify kinetic parameters associated with PDH, GAPDH, and CS as the primary determinants of τ_{max} across the network. However, they are not the highest “hits” in steady state 1, and other parameters have high occurrence in individual steady states.

The reviewer is correct that the kinetic parameters associated with PDH, GAPDH, and CS do not appear as the highest-occurrence hits in steady state 1. As shown in Figure 3a and Supplementary

Figure 10, there is substantial variability in the occurrence of individual enzymes across different steady states. These occurrences depend strongly on the specific steady-state fluxes and metabolite concentrations, which naturally fluctuate across the steady states we sampled.

However, the goal of our analysis was not to identify enzymes that dominate *within a single steady state*, but rather to detect enzymes that recur consistently *across multiple steady states*. Such a recurring occurrence is particularly valuable because it improves generalizability and reduces uncertainty in the modelling process. When we tested these recurring enzymes for their influence on the system's dynamic properties, we found that they indeed controlled the system's output across multiple steady-state conditions, thereby validating their network-level importance, despite not always being top hits in each steady state.

Minor concerns

- The methods related to sampling "four different thermodynamically feasible reference profiles of fluxes and metabolite concentrations using Thermodynamic Flux Balance Analysis" were not clear. The text in the Methods section seemed incomplete and some of it read as if generated by an LLM (referring to a "study titled" did not make sense).
We have revised the Methods section to include additional explanations and clarifications.

- Figure 5c: "Left" and "right" are switched. There is a reference to text in Figure 5d that is not present.
We thank the reviewer for bringing these errors to our attention. The "left" and "right" labels in Figure 6c (formerly Figure 5c) have been corrected, and the erroneous reference in the caption of Figure 6d (formerly Figure 5d) has been removed. The caption of Figure 6 (formerly Figure 5) has also been revised for clarity.

References

(1) Saltelli, A. Making Best Use of Model Evaluations to Compute Sensitivity Indices. *Comput. Phys. Commun.* **2002**, *145* (2), 280–297. https://doi.org/10.1016/S0010-4655(02)00280-1.
(2) Saltelli, A.; Annoni, P.; Azzini, I.; Campolongo, F.; Ratto, M.; Tarantola, S. Variance Based Sensitivity Analysis of Model Output. Design and Estimator for the Total Sensitivity Index. *Comput. Phys. Commun.* **2010**, *181* (2), 259–270. https://doi.org/10.1016/j.cpc.2009.09.018.

Authors response to the reviewers' comments

Title: Generative Approaches to Kinetic Parameter Inference in Metabolic Networks via Latent Space Exploration
Tracking #: NCOMMS-25-56564B
Authors: Choudhury et al.

We thank the Editor and the reviewers for the time and care invested throughout the review process.

Our point-by-point responses to the reviewers' comments are provided below in blue.

REVIEWERS' COMMENTS

Reviewer #1 (Remarks to the Author):

Thank you for addressing my comments.

Additional minor comment: Scripts for Figure 6 on the GitHub page was still labelled Figure 5.

Thank you for catching this. We have corrected the figure numbering in the GitHub repository: the relevant scripts now correctly refer to Figure 6.

Reviewer #1 (Remarks on code availability):

Codes have README file.
I did not try to run the code as I do not have GPU capability on my machine.

Thank you for reviewing the code availability. We have clarified the hardware requirements in the README, explicitly noting that our latent space exploration method and RENAISSANCE run on CPUs, whereas GPUs are recommended for training generative adversarial networks in REKINDLE.

Reviewer #2 (Remarks to the Author):

The authors have improved the manuscript in revision and have adequately addressed my concerns.

We thank the reviewer for their careful assessment and for confirming that the revision adequately addresses their concerns.
